# Proteomic profiling of cellular steatosis with concomitant oxidative stress in vitro

**DOI:** 10.1186/s12944-016-0283-7

**Published:** 2016-07-02

**Authors:** Khalida Ann Lockman, Varanand Htun, Rohit Sinha, Philipp Treskes, Leonard J. Nelson, Sarah F. Martin, Sophie M. Rogers, Thierry Le Bihan, Peter C. Hayes, John N. Plevris

**Affiliations:** Hepatology Laboratory, University of Edinburgh, 49 Little France Crescent, Edinburgh, EH16 4SB Scotland, UK; Kinetic Parameter Facility, SynthSys - Centre for Synthetic and Systems Biology, University of Edinburgh, Edinburgh, EH9 3BF UK

**Keywords:** Nonalcoholic fatty liver, Steatohepatitis, Oxidative stress

## Abstract

**Background:**

Nutrient excess underpins the development of nonalcoholic fatty liver disease (NAFLD). The ensuing metabolic derangement is characterised by increased cellular respiration, oxidative stress and mitochondrial impairment. We have previously recapitulated these events in an in vitro cellular steatosis model. Here, we examined the distinct patterns of protein expression involved using a proteomics approach.

**Methods:**

Human hepatoblastoma C3A cells were treated with a combination of energy substrates; lactate (L), pyruvate (P), octanoate (O) and ammonia (N). Proteins extracts were trypsinized and analyzed on a capillary HPLC OrbitrapXL mass spectrometer. Proteins were quantified using a label-free intensity based approach. Functional enrichment analysis was performed using ToppCluster via Gene Ontology (GO) database.

**Results:**

Of the 1327 proteins identified, 104 were differentially expressed between LPON and untreated cells (defined as: ≥2 peptides; fold change ≥1.5; *p*-value <0.05). Seventy of these were upregulated with LPON. Functional enrichment analysis revealed enhanced protein biosynthesis accompanied by downregulation of histones H2A type 1-A, H1.2, H1.5 and H1.0I in LPON cells. Lipid binding annotations were also enriched as well as proteins involved in cholesterol synthesis, uptake and efflux. Increased expression of aldo-keto reductase family 1, member C1 and C3 suggests enhanced sterol metabolism and increased ROS-mediated lipid peroxidation.

**Conclusions:**

The surge of energy substrates diverts free fatty acid metabolism towards pathways that can mitigate lipotoxicity. The histones depletion may represent an adaptation to increased protein synthesis. However, this can also expose DNA to oxidative stress thus should be explored further in the context of NAFLD progression.

## Background

Nonalcoholic fatty liver disease (NAFLD) is becoming increasingly prevalent globally, often presenting alongside Type 2 diabetes and obesity [1]. According to a recent study, the prevalence of NAFLD in a middle-aged population living in the United States is 46 % and of those, 29.9 % were discovered to have progressed to nonalcoholic steatohepatitis (NASH) [2]. Although traditionally thought of as a western disease, the prevalence of NAFLD is now also high in Asia; as high as 45 % in parts of south Asia and East Asia [3].

NAFLD is characterized by intracellular triglyceride accumulation within the liver that potentially, in the presence of inflammation, can progress to NASH and eventually can lead to liver cirrhosis [4, 5]. Increased free fatty acids (FFA) delivery to the liver is the crux of NAFLD development. A dietary surge of FFAs can lead to triglyceride accumulation and, importantly, it can enhance mitochondrial respiration thus increasing the formation of reactive oxygen species (ROS) [6, 7]. A prominent theory is that the progression from steatosis to steatohepatitis and cirrhosis in part, is caused by hepatic lipotoxicity. The toxicity effects appear to stem from FFA rather than steatosis per se [8]. Both FFAs and their metabolites have been implicated in the induction of inflammation and apoptosis via an increase in ROS, and by activating signalling pathways that can precipitate apoptosis [9, 10].

The precise molecular mechanisms are yet to be elucidated but are likely to involve complex interactions between various intra- and extracellular metabolic signalling networks. Recent advances in proteomics can potentially unravel these relevant pathways. The systematic protein profiling achieved by proteomics can identify distinct patterns of protein expression in response to the metabolic derangement in NAFLD. There have been recent proteomics in vivo studies set to identify suitable markers of NAFLD progression as well as examining protein expression in serum samples and liver tissue from patients with NAFLD [11, 12]. However, these studies are not geared to differentiate events that occur specifically within the hepatocyte from those that have arisen peripherally.

We have previously shown that C3A hepatoblastoma cells treated with combinations of physiological energy substrates; lactate (L), pyruvate (P), octanoate (O) and ammonia (N) in vitro recapitulate the events that have been proposed to occur in NAFLD [13]. The combination of these substrates induces significant steatosis. Importantly, these substrates synergize to enhance tricarboxylic acid cycle (TCA) activity hence increased mitochondrial respiration. The resultant rise in ROS culminates in morphological and functional alterations of the hepatic mitochondria. Thus, LPON-induced cellular steatosis manifests many of the key features associated with steatohepatitis such as impaired mitochondrial function, oxidative stress, and altered glucose metabolism. In this study, using a high-resolution focused proteomics approach, we examined distinct patterns of protein expression in our vitro model of cellular steatosis.

## Methods

### Cell culture

Human hepatoblastoma C3A cells (CRL-10741, American Type Culture Collection, Manassas, VA, USA) were cultured in 75 cm^2^ tissue culture flasks in minimal essential medium Eagle (MEME) (Sigma, Poole, UK, cat. no. M0268) with 10 % fetal calf serum (FCS) (Gibco, Life Technologies, CA, USA, cat. no. 10270) and antibiotics (100 IU/ml penicillin, 100 mg/ml streptomycin) (Sigma, Poole, UK, cat. no. P0781) at 37 °C and 5 % CO_2_ until 70 % confluent. The medium was changed every 48 h after initial plating. Cells were passaged every 7–10 days. MEME with FCS in the experimental plates was supplemented with combinations of octanoate (O) (2 mM) (Sigma, Poole, UK, cat. no. C2875), lactate (L) (10 mM) (Sigma, Poole, UK, cat. no. L7022), pyruvate (P) (1 mM) (Sigma, Poole, UK, cat. no. P5280) and ammonia (N) (4 mM), (Sigma, Poole, UK, cat. no. A4514). Cells were cultured in the supplemented media for 72 h prior to experimentation.

### Sample preparation

Supernatant from each treatment group was aspirated, and the cell monolayer was rinsed twice with 2 ml of phosphate buffered saline (PBS) (Sigma, Poole, UK, cat. no. D8662) (with added calcium chloride and magnesium chloride). Further PBS (500 ml) was added to each well and cells were then gently harvested. The pellets were kept frozen at −80 °C until proteomics analysis. For analysis, samples were centrifuged, supernatant removed and cells were resuspended in 65 μl urea 8 M (Sigma, Poole, UK, cat. no. U1250). ‬‬ Protein concentration was quantified using Bradford protein assay (Bio-Rad, Hertfordshire, UK, cat. no. 500-0205). Cell pellets (~200 μg protein) were lysed with dithiothreitol (DTT) 5 mM (Melford Laboratories, Ipswich, UK, cat. no. MB1015), reduced, alkylated with iodoacetamide 10 mM (Sigma, Poole, UK, cat. no. I1149), digested with 8 μg trypsin, L-1-Tosylamide-2-phenylethyl chloromethyl ketone (TPCK) treated, (Worthington Biochem, Lorne Laboratories, Reading, UK, Cat. No. LS003750) overnight at room temperature. A total of 4 μg peptides were cleaned on ZipTip (Millipore, Hertfordshire, UK, Cat. No. ZTC18S096) reverse phase tips and 2 μg were loaded on a Capillary-HPLC-MS. Capillary-HPLC-MS/MS analysis was done using a system consisting of a micro-pump (1200 binary HPLC system, Agilent, UK) coupled to a hybrid LTQ-Orbitrap XL instrument (Thermo-Fisher, UK). Data were quantified label-free using Progenesis LC-MS in combination with MASCOT search engine and the Ref_HSapiens protein database in a similar manner as published previously (14).

### Bioinformatics

Data were quantified label-free as described previously [14, 15]. Multicharged (2+,3+,4+) ion intensities were extracted from LC-MS files and MSMS data were searched using Mascot Version 2.4 (Matrix Science Ltd, UK) against the *Homo Sapiens* subset of the NCBI protein database (34,281 sequences) using a maximum missed-cut value of 2, variable oxidation (M), N-terminal protein acetylation and fixed carbamidomethylation (C); precursor mass tolerance was 7 ppm and MSMS tolerance 0.4 Da. The significance threshold (*p*) was < 0.05 (MudPIT scoring). A minimum peptide cut off score of 20 was set, corresponding to <3 % global false discovery rate (FDR) using a decoy database search. Proteins identified and quantified with two or more peptide sequences were retained.

### Enrichment analysis

Over represented molecular functions and biological processes in LPON treated and untreated C3A cells were identified by ToppCluster via Gene Ontology (GO) database (https://toppcluster.cchmc.org/) [16]. Enriched functional annotations of this proteomics dataset were identified under GO: molecular function, GO: biological process, and GO: cellular component. Functional annotations with a *p*-Value cutoff of *p* < 0.05 with Bonferroni correction were considered. Gene limits were set to default 1 ≤ *n* ≤ 1500. Next, proteins were identified using BLAST search to identify homologous protein sequences from The National Center for Biotechnology Information (NCBI) database (www.ncbi.nlm.nih.gov). InterPro terms were also integrated with BLAST-derived GO terms. Enrichment analysis was then performed again using Blast2GO [17]. Category 4 proteins (Table 1) were used as the background set. Functional annotations with a p-value cutoff of *p* < 0.05 and FDR correction <0.05 were considered. All other settings were kept at default.

**Table 1:** Summary of all identified proteins from proteomics profiling

Protein category	Quantified peptides	*P*-value (ANOVA)	Number of proteins	Ratio between conditions
1	>2	<0.05	104	>1.5 fold
2	1	<0.05	98	>1.5 fold
3	>1	<0.05	229	<1.5 fold
4	>1	>0.05	896	Not applicable

Proteins identified were divided into 4 categories based on the quantity of peptides, ratio between conditions and *p*-value. Differential expression of proteins between LPON and the untreated cells was considered as significant if fold-change was ≥1.5, quantified with at least two peptides and *p*-value <0.05 (Category 1)

### Statistical analysis

A two-tailed t-test for independent samples or biological triplicates was performed on ArcSinh transformed, normally distributed intensity data. Proteins with *p* < 0.05 and absolute mean changes of 1.5-fold or larger were considered significant.

## Results

Findings from the proteomics profiling divided into four categories are summarized in Table 1. Of the 1327 proteins identified, 104 of these met the criteria for Category 1 in Table 1 (defined as: fold change ≥1.5; quantified with at least two peptides; *p*-value <0.05) and were considered significant between LPON and the untreated group.

Of the Category 1 proteins, 70 (67.3 %) were significantly upregulated in LPON treated cells (Table 2). Protein expression in untreated cells were taken to be the baseline. Hence, a protein was deemed as downregulated in LPON treated cells when its expression met the criteria for Category 1 (Table 1) and was greater in untreated cells than that seen in LPON treated cells. This is demonstrated in Table 3, which shows the remaining 34 Category 1 proteins (32.7 %) that were expressed in lower abundance with LPON when compared with the untreated group.

**Table 2:** Significantly upregulated proteins in LPON treated cells

Accession	Description	HGNC symbol	Peptide count	Confidence score	*P*-value	Fold change from control
NP_000468.1	serum albumin preproprotein	ALB	4	173.4	0.00	11.33
NP_001113.2	perilipin-2	PLIN2	6	387.7	0.02	10.42
NP_000030.1	apolipoprotein A-I preproprotein	APOA1	2	107.5	0.02	4.08
NP_004453.3	squalene synthase	FDFT1	3	159.4	0.02	3.22
NP_005327.1	vigilin	HDLBP	3	137.1	0.00	2.72
NP_005902.1	S-adenosylmethionine synthase isoform type-2	MAT2A	3	195.1	0.00	2.50
NP_001344.2	aldo-keto reductase family 1 member C1	AKR1C1	4	226.8	0.00	2.41
NP_006627.2	bifunctional methylenetetrahydrofolate dehydrogenase/cyclohydrolase, mitochondrial precursor	MTHFD2	2	59.6	0.02	2.39
NP_009224.2	complement C4-A preproprotein	C4A	3	119.5	0.01	2.36
NP_006382.1	importin-7	IPO7	2	55.6	0.01	2.35
NP_036205.1	T-complex protein 1 subunit epsilon	CCT5	4	255.7	0.00	2.30
NP_001407.1	eukaryotic initiation factor 4A-I	EIF4A1	7	314.6	0.00	2.28
NP_000032.1	apolipoprotein E precursor	APOE	3	152.7	0.00	2.21
NP_001054.1	serotransferrin precursor	TF	6	369.9	0.02	2.18
NP_001165908.1	retrotransposon-derived protein PEG10 isoform 3	PEG10	2	185.2	0.01	2.13
NP_149351.1	surfeit locus protein 4	SURF4	2	251.8	0.01	2.13
NP_002769.1	proactivator polypeptide isoform a preproprotein	PSAP	4	243.4	0.00	2.09
NP_001952.1	elongation factor 2	EEF2	13	713.0	0.00	2.07
NP_055121.1	hypothetical protein LOC51493	RTCB	2	88.1	0.01	2.05
NP_001434.1	fatty acid-binding protein, liver	FABP1	4	329.0	0.02	2.01
NP_110379.2	T-complex protein 1 subunit alpha isoform a	TCP1	6	392.6	0.01	2.01
NP_056348.2	bifunctional ATP-dependent dihydroxyacetone kinase/FAD-AMP lyase (cyclizing)	TKFC	3	169.5	0.03	2.00
NP_000508.1	hemoglobin subunit alpha	HBA2	2	80.4	0.01	1.99
NP_001159757.1	T-complex protein 1 subunit eta isoform d	CCT7	2	194.3	0.01	1.93
NP_000448.3	hepatocyte nuclear factor 4-alpha isoform b	HNF4A	2	109.1	0.00	1.92
NP_001753.1	T-complex protein 1 subunit zeta isoform a	CCT6A	5	408.0	0.01	1.92
NP_001650.1	ADP-ribosylation factor 3	ARF3	3	268.5	0.00	1.90
NP_006364.2	synaptic vesicle membrane protein VAT-1 homolog	VAT1	3	174.1	0.01	1.89
NP_006262.1	protein S100-A1	S100A1	2	215.4	0.02	1.88
NP_000867.2	cation-independent mannose-6-phosphate receptor precursor	IGF2R	3	103.2	0.02	1.88
NP_000383.1	canalicular multispecific organic anion transporter 1	ABCC2	2	104.3	0.05	1.86
NP_001596.2	alanyl-tRNA synthetase, cytoplasmic	AARS	3	150.6	0.02	1.81
NP_002286.2	40S ribosomal protein SA	RPSA	3	194.7	0.00	1.80
NP_001171669.1	src substrate cortactin isoform c	CTTN	2	62.4	0.01	1.80
NP_005882.2	acetyl-CoA acetyltransferase, cytosolic	ACAT2	3	159.6	0.02	1.79
NP_001016.1	40S ribosomal protein S23	RPS23	2	63.0	0.00	1.76
NP_056174.2	zinc transporter ZIP14 isoform b	SLC39A14	3	178.6	0.02	1.76
NP_003730.4	aldo-keto reductase family 1 member C3	AKR1C3	3	257.7	0.02	1.73
NP_005971.1	protein S100-P	S100P	3	164.9	0.02	1.72
NP_002622.2	6-phosphogluconate dehydrogenase, decarboxylating	PGD	5	502.0	0.02	1.70
NP_005821.2	28S ribosomal protein S31, mitochondrial precursor	MRPS31	2	78.2	0.00	1.68
NP_001012.1	40S ribosomal protein S17	RPS17	5	349.2	0.01	1.68
NP_006422.1	T-complex protein 1 subunit beta isoform 1	CCT2	5	481.5	0.03	1.68
NP_001018146.1	NME1-NME2 protein	NME1	2	105.5	0.00	1.67
NP_004035.2	bifunctional purine biosynthesis protein PURH	ATIC	5	247.7	0.00	1.67
NP_038203.2	isoleucyl-tRNA synthetase, cytoplasmic	IARS	2	95.1	0.02	1.65
NP_000775.1	sterol 26-hydroxylase, mitochondrial precursor	CYP27A1	2	130.2	0.03	1.64
NP_006545.1	metaxin-2	MTX2	2	127.2	0.00	1.64
NP_002203.1	eukaryotic translation initiation factor 6 isoform a	EIF6	3	267.9	0.00	1.64
NP_006089.1	guanine nucleotide-binding protein subunit beta-2-like 1	GNB2L1	5	233.4	0.00	1.64
NP_001961.1	eukaryotic translation initiation factor 5A-1 isoform B	EIF5A	4	164.5	0.04	1.64
NP_631961.1	TATA-binding protein-associated factor 2 N isoform 1	TAF15	2	126.7	0.00	1.63
NP_001002.1	40S ribosomal protein S7	RPS7	3	178.1	0.00	1.62
NP_004387.1	probable ATP-dependent RNA helicase DDX5	DDX5	3	145.9	0.02	1.61
NP_036555.1	60S ribosomal protein L13a	RPL13A	2	72.2	0.00	1.61
NP_000996.2	40S ribosomal protein S3	RPS3	6	264.7	0.01	1.60
NP_006752.1	14-3-3 protein epsilon	YWHAE	3	221.6	0.00	1.60
NP_055205.2	staphylococcal nuclease domain-containing protein 1	SND1	12	567.8	0.00	1.58
NP_001000.2	40S ribosomal protein S5	RPS5	3	266.6	0.01	1.57
NP_005557.1	L-lactate dehydrogenase A chain isoform 1	LDHA	6	527.9	0.01	1.57
NP_005337.2	heat shock 70 kDa protein 1A/1B	HSPA1B	8	517.1	0.01	1.57
NP_061819.2	sialic acid synthase	NANS	3	152.4	0.01	1.56
NP_001419.1	alpha-enolase	ENO1	14	1032.9	0.02	1.56
NP_733779.1	S-phase kinase-associated protein 1 isoform b	SKP1	3	123.7	0.01	1.55
NP_002385.3	4 F2 cell-surface antigen heavy chain isoform c	SLC3A2	11	680.1	0.03	1.53
NP_001279.2	chloride intracellular channel protein 1	CLIC1	4	251.6	0.00	1.53
NP_000792.1	peptidyl-prolyl cis-trans isomerase FKBP1A isoform a	FKBP1A	2	126.4	0.01	1.52
NP_001393.1	elongation factor 1-alpha 1	EEF1A1	16	996.3	0.00	1.52
NP_003893.2	far upstream element-binding protein 1	FUBP1	2	165.0	0.00	1.52
NP_006392.1	acidic leucine-rich nuclear phosphoprotein 32 family member B	ANP32B	2	149.5	0.02	1.50

Of the 104 proteins that met the criteria for Category 1, 70 were significantly upregulated with LPON

**Table 3:** Significantly upregulated proteins in untreated cells

Accession	Description	HGNC symbol	Peptide count	Confidence score	*P-*value	Fold change from LPON
NP_734466.1	histone H2A type 1-A	HIST1H2AA	5	321.3	0.02	4.63
NP_005310.1	histone H1.2	HIST1H1C	11	734.0	0.02	3.30
NP_057395.1	ATPase inhibitor, mitochondrial isoform 1 precursor	ATPIF1	2	53.7	0.05	3.01
NP_002404.1	microsomal glutathione S-transferase 2	MGST2	2	61.3	0.03	2.47
NP_061849.2	sodium-coupled neutral amino acid transporter 2	SLC38A2	2	281.4	0.02	2.34
NP_115520.2	ras-related protein Rab-6C	RAB6C	2	78.7	0.04	2.30
NP_000021.1	serine--pyruvate aminotransferase	AGXT	2	103.1	0.01	2.03
NP_112487.1	SRA stem-loop-interacting RNA-binding protein, mitochondrial precursor	SLIRP	2	118.7	0.04	1.99
NP_003283.2	nucleoprotein TPR	TPR	3	244.9	0.01	1.91
NP_004233.1	high mobility group nucleosome-binding domain-containing protein 3 isoform HMGN3a	HMGN3	2	122.5	0.00	1.89
NP_005313.1	histone H1.5	HIST1H1B	6	464.7	0.02	1.86
NP_001070956.1	protein ALEX isoform f	GNAS	3	104.8	0.02	1.81
NP_001600.1	short/branched chain specific acyl-CoA dehydrogenase, mitochondrial precursor	ACADSB	7	517.8	0.01	1.76
NP_056230.1	brain protein 44	MPC2	2	88.0	0.04	1.76
NP_005361.2	ras-related protein Rab-8A	RAB8A	2	95.4	0.01	1.71
NP_002148.1	10 kDa heat shock protein, mitochondrial	HSPE1	5	356.0	0.00	1.70
NP_006852.1	ras-related protein Rab-35 isoform 1	RAB35	2	92.2	0.03	1.70
NP_003365.1	voltage-dependent anion-selective channel protein 1	VDAC1	8	807.2	0.01	1.68
NP_001139021.1	thioredoxin domain-containing protein 5 isoform 3	TXNDC5	6	387.9	0.02	1.66
NP_002130.2	heterogeneous nuclear ribonucleoprotein G isoform 1	RBMX	11	509.6	0.00	1.63
NP_005349.3	LIM domain only protein 7 isoform 1	LMO7	6	317.6	0.04	1.62
NP_079034.3	agmatinase, mitochondrial precursor	AGMAT	5	338.0	0.00	1.62
NP_002734.2	glucosidase 2 subunit beta isoform 1	PRKCSH	10	506.0	0.00	1.60
NP_057318.2	long-chain-fatty-acid--CoA ligase 5 isoform a	ACSL5	10	561.8	0.01	1.59
NP_005309.1	histone H1.0	H1F0	6	380.0	0.03	1.59
NP_001059.2	DNA topoisomerase 2-beta	TOP2B	2	107.4	0.01	1.57
NP_002851.2	delta-1-pyrroline-5-carboxylate synthase isoform 1	ALDH18A1	20	1184.8	0.00	1.57
NP_006293.2	mannosyl-oligosaccharide glucosidase isoform 1	MOGS	4	173.2	0.01	1.57
NP_059980.2	transmembrane emp24 domain-containing protein 9 precursor	TMED9	3	182.6	0.01	1.57
NP_065737.2	4-aminobutyrate aminotransferase, mitochondrial precursor	ABAT	11	699.1	0.00	1.57
NP_006017.1	histone H1x	H1FX	3	195.5	0.04	1.55
NP_055866.1	endoplasmic reticulum resident protein 44 precursor	ERP44	2	153.0	0.05	1.54
NP_056240.2	DNA-directed RNA polymerase I subunit RPA1	POLR1A	2	42.2	0.01	1.51
NP_057371.2	heterochromatin protein 1-binding protein 3	HP1BP3	4	221.1	0.02	1.51

Thirty-four proteins in Category 1 were significantly upregulated in untreated cells

Significantly enriched GO: Molecular Functions of proteins upregulated with LPON (Table 2) and proteins downregulated with LPON (Table 3) derived from ToppCluster are summarized in Tables 4 and 5 respectively.

**Table 4:** Summary of enriched GO: Molecular Function terms in LPON treated cells

Enriched GO: molecular function terms	GO database ID	Log of *p*-value	Proteins enriched in set
structural constituent of ribosome	GO:0003735	10	MRPS31 RPL13A RPS17 RPS23
			RPS3 RPS5 RPS7 RPSA
poly(A) RNA binding	GO:0044822	10	CCT6A DDX5 EEF1A1 EEF2 EIF4A1
			EIF5A ENO1 FUBP1 GNB2L1 HDLBP
			HSPA1B MRPS31 NME1 PEG10 RPL13A
			RPS17 RPS23 RPS3 RPS5 RPS7RPSA
			RTCB SLC3A2 SND1 TAF15 TCP1 YWHAE
unfolded protein binding	GO:0051082	5.5252	CCT2 CCT5 CCT6A CCT7 HSPA1B TCP1
carboxylic acid binding	GO:0031406	5.51	AARS AKR1C1 ALB FABP1
			HNF4A IGF2R MAT2A YWHAE
organic acid binding	GO:0043177	5.4964	AARS AKR1C1 ALB FABP1 HNF4A
			IGF2R MAT2A YWHAE
monocarboxylic acid binding	GO:0033293	5.1232	AKR1C1 ALB FABP1
			HNF4A IGF2R
translation factor activity, RNA binding	GO:0008135	4.5729	EEF1A1 EEF2 EIF4A1 EIF5A EIF6
ketosteroid monooxygenase activity	GO:0047086	4.3661	AKR1C1 AKR1C3
indanol dehydrogenase activity	GO:0047718	4.3661	AKR1C1 AKR1C3
androsterone dehydrogenase activity	GO:0047023	4.0662	AKR1C1 AKR1C3
trans-1,2-dihydrobenzene-1,2-diol dehydrogenase activity	GO:0047115	4.0662	AKR1C1 AKR1C3
17-alpha,20-alpha-dihydroxypregn-4-en-3-one dehydrogenase activity	GO:0047006	4.0662	AKR1C1 AKR1C3
translation elongation factor activity	GO:0003746	3.9915	EEF1A1 EEF2 EIF5A

Overrepresented GO: Molecular Function terms were identified by ToppCluster. HGNC symbol is used to display proteins enriched under the GO term. *p*-value cutoff for enriched terms was <0.05 with Bonferroni correction applied to minimize false-positive detection

**Table 5:** Summary of enriched GO: molecular function terms in untreated cells

Enriched GO: molecular function terms	GO database ID	Log of *P*-value	Proteins enriched in set
chromatin DNA binding	GO:0031490	4.9613	H1F0 HIST1H1B HIST1H1C HMGN3
chromatin binding	GO:0003682	4.9031	H1F0 HIST1H1B HIST1H1C
			HMGN3 RBMX TOP2B TPR
poly(A) RNA binding	GO:0044822	3.7078	ALDH18A1 H1F0 H1FX HIST1H1B
			HIST1H1C HSPE1 RBMX SLIRP TPR

Overrepresented GO: Molecular Function terms were detected by ToppCluster. *P*-value cutoff for enriched terms was *p*-value < 0.05, in the table this is presented of Log of *p*-value. Terms included has *p*-values subjected to Bonferroni correction to minimize false-positive detection. HGNC symbol is used to display proteins enriched under the GO term

### LPON treated cells

Proteins involved in lipid metabolism were increased with LPON as evident by GO: molecular function annotations enriched in response to LPON treatment such as ‘carboxylic acid binding’, ‘organic acid binding’ and ‘monocarboxylic acid binding’ (Table 4). Common upregulated proteins within these annotations were serum albumin preprotein (ALB; fold change: 11.33), aldo-keto reductase family 1 member C1 (AKR1C1; fold change: 2.41), fatty acid binding protein 1 (FABP1; fold change: 2.01), hepatocyte nuclear factor 4-alpha (HNF4A; fold change: 1.92), and insulin-like growth factor 2 receptor (IGF2R; fold change: 1.92) (Table 2).

**Fig. 1:**
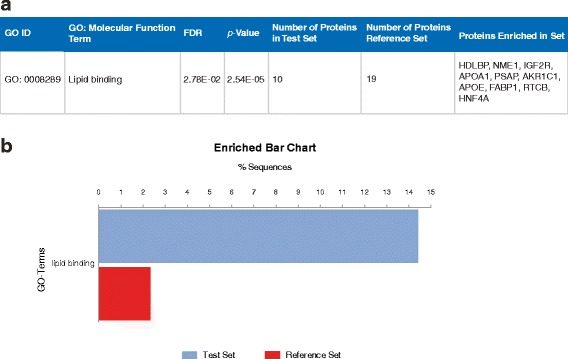
Enrichment analysis for LPON treated cells using Blast2GO. **a** Overrepresented GO: Molecular Function terms in LPON treated cells. **b** Enrichment bar chart of significant GO: Molecular Function term demonstrating the percentage of sequences annotated with LPON compared with the reference set based on the Fishers Exact Test results

Enrichment analysis by Blast2GO (Fig. 1) found only “lipid binding”, a broader GO term, to be enriched. Proteins found to be enriched in this set, that were not found by ToppCluster, included apolipoprotein A-I preproprotein (APOA1; fold change: 4.08), vigilin (HDLBP; fold change: 2.72), and apolipoprotein E precursor (APOE; fold change: 2.21).

However, several noteworthy upregulated proteins involved in lipid processing were not found in the enriched GO: Molecular Function annotations in Table 4 or Fig. 1. These were perilipin-2 (PLIN2; fold change: 10.42), squalene synthase (FDFT1; fold change: 3.22) and sterol 27-hydroxylase (CYP27A1; fold change: 1.64) (Table 2). These proteins are included under enriched GO: biological process annotations ‘lipid localisation’, ‘lipid transport’ and ‘steroids metabolism’.

Three significant GO: Molecular Function annotations implicating in increase protein synthesis in response to LPON are ‘translation factor activity, RNA binding’, ‘translation elongation factor activity’, and ‘unfolded protein binding’ (Table 4). A protein of interest included in the former two terms is elongation factor 1-alpha 1 (EEF1A1; fold change: 1.52) (Table 2). Whilst the latter term implicates the components of the TCP-1 Ring Complex (TRiC) by including T-complex protein 1 subunit alpha isoform a (TCP1; fold change: 2.01) T-complex protein 1 subunit beta isoform 1 (CCT2; fold change: 1.68), T-complex protein 1 subunit epsilon (CCT5; fold change: 2.30), T-complex protein 1 subunit zeta isoform a (CCT6A; fold change: 1.92) and T-complex protein 1 subunit eta isoform d (CCT7; fold change: 1.93) (Table 2).

### Untreated cells

**Fig. 2:**
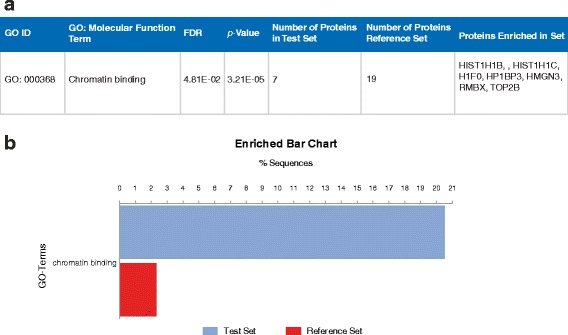
Enrichment analysis for untreated cells using Blast2GO. **a** Overrepresented GO: Molecular Function terms in untreated cells. **b** Enrichment bar chart of significant GO: Molecular Function term demonstrating the percentage of sequences annotated compared with the reference set based on the Fishers Exact Test results

‘Chromatin binding’ and its more specific child term ‘chromatin DNA binding’ were both enriched in untreated cells (Table 5). In support of this, enrichment analysis by Blast2GO (Fig. 2) also found the broader GO term, ‘chromatin binding’ to be significantly enriched. Although proteins enriched in the set varied slightly, common proteins found in these three sets are as follows: histone H1.0 (H1F0; fold change: 1.59), histone H1.5 (HIST1H1B; fold change: 1.86), histone H1.2 (HIST1H1C; fold change: 3.30), heterochromatin protein 1-binding protein (HMGN3; fold change: 1.89). Common proteins found in these two related terms are as follows: histone H1.0 (H1F0; fold change: 1.59), histone H1.5 (HIST1H1B; fold change: 1.86), histone H1.2 (HIST1H1C; fold change: 3.30), heterochromatin protein 1-binding protein (HMGN3; fold change: 1.89). Although not included in the aforementioned annotations, other histone H1 proteins were also downregulated in LPON-treated cells whilst histone H2A type 1-A (HIST1H2AA; fold change: 4.63) was also found to be the most downregulated (Table 3).

## Discussion

The advancement of proteomics has unquestionably provided important insight into the pathogenesis of human NAFLD. We applied this technique to an in vitro model of cellular steatosis designed to recapitulate the effects of excessive energy substrates. Our data highlight several relevant protein changes that occur in the context of intracellular triglyceride accumulation.

### Increased fatty acid processing

Proteins involved in lipid metabolism were increased in the presence of free fatty acids. The rise in albumin (11-fold) and the abundance of FABP1 suggest enhanced free fatty acid sequestering/uptake. Indeed, the binding of free fatty acids to albumin and FABP1 is crucial for preventing lipotoxicity [8]. However, in contrast to our current study, FABP1 has been found to be down regulated in patients with NAFLD as well as in animal models [18]. Although in this study (18) FABP1 was induced by HNF4A expression, it is also important to note that FABP1 expression in their mice model of NAFLD, via methionine and choline deficient diet, decreased gradually over a period of 5 weeks. Likewise, in humans, NAFLD development is often a slow and insidious process. Another study discovered that FABP1, in human samples, was overexpressed in simple steatosis and under expressed in NASH tissue samples [19].

Similar to that seen in human and rodent NAFLD, triglyceride accumulation in the LPON model is paralleled by an upregulation of perilipin-2 (PLIN2) [20]. PLIN2 was the second-most upregulated protein (10-fold) in response to LPON. Although its precise role in fatty acid metabolism is yet to be determined. PLIN2 expression is thought to reduce lipolysis hence decreasing the overall triglyceride turnover [21]. It is possible that this represents a protective mechanism against the deleterious effects of FFA. In support of this, a study has shown that in the absence of PLIN2, perilipin-5 was upregulated to enhance fatty acid oxidation and to protect mitochondria from oxidative stress induced by fatty acids [22].

The rationale for using octanoate in this study is that it accelerates β-oxidation resulting in a swift formation of acetyl-coA akin to that seen with the surge of energy substrates in human dietary NAFLD. The fate of the superfluous acetyl-coA is crucial in the pathophysiology of NAFLD. If the flux into TCA cycle is exceeded, acetyl-coA would be diverted towards non-oxidative pathways including the mevalonate pathway for cholesterol and steroid synthesis. In LPON treated cells, the diversion of acetyl-coA towards mevalonate pathway is evident by a 3.2-fold increase in the expression of squalene synthase, the first committed step to sterol synthesis. Furthermore, the significant rise in the expression of apolipoprotein A1 (APOA1), high density lipoprotein binding protein (HDLBP) and apolipoprotein E (APOE), all of which were included in the enriched GO: biological process ‘lipid transport’ supports enhanced cholesterol efflux to meet the demand of increased synthesis. Hepatic cholesterol synthesis has been shown to be upregulated in human NAFLD and its concentration correlates with disease severity [23].

### Increase in protein synthesis and evidence for Endoplasmic Reticulum (ER) stress

LPON treatment enriches proteins involved in protein biosynthesis. Given the up regulation of many other proteins in our dataset in response to increased metabolic demand, the concurrent upregulation for translation elongation factors that facilitates formation of peptide bonds is expected. Increased expression of TCP-1 Ring Complex (TRiC) constituents namely chaperon containing TCP1 (CCT) proteins in response to LPON is also suggestive of increase protein synthesis. However, it should be noted that a study on different cell lines, including HepG2, found that cancer cell lines tended to overexpress TRiC proteins and that expression levels did not necessarily correlate with TRiC activity [24].

Proteins, however, often have more than one function. eEF1A1, one of the enriched proteins under the annotation ‘Translation Elongation Factor Activity’, may also have a role in modulating lipotoxicity. eEF1A1 expression was found to be induced in HepG2 cells in response to palmitic acid as well as in obese mice with severe hepatic steatosis and ER stress [25]. ER stress is caused by an accumulation of unfolded proteins in the ER lumen; this initiates the unfolded protein response (UPR) potentially leading to cell death. Although the precise role of eEF1A1 in mediating lipotoxic cell death is not known, it is thought eEF1A1 alters the actin cytoskeleton leading to cell death in response to ER stress caused by increased ROS production [26].

Protein synthesis is complex. It involves posttranslational modifications, which are not examined in this present study. Further, it is an anabolic process that requires ATP. We have previously shown that chronic LPON treatment diminished mitochondrial respiration [13]. It is unknown how this would affect ATP formation in these cells. The finding of decreased expression of ATPase inhibitor certainly suggests that the cellular demand for ATP is met. The endogenous ATPase inhibitor is thought to conserve ATP by limiting the consumption of cellular ATP in the presence of significant mitochondrial impairment [27].

### Possible mechanism of injury

We have previously shown that LPON treatment resulted in increased ROS formation [13]. It is likely that increased ROS formation with LPON modulates protein expression such that cellular defense against oxidative/electrophilic stress including those from lipid peroxidation can be mounted. The expression of AKR1C1 was raised in the LPON model. This is in concordance with other studies that have shown AKR1C1 to be upregulated in response to ROS production. Further, it is known to reduce byproducts of lipid peroxidation such as α, β-unsaturated aldehydes [28]. AKR1C1 upregulation is also associated with increased transcription factor NF-E2-related factor-2 (NRF2) activity, which plays a crucial role in the upregulation of antioxidant pathways [29].

Oxidative stress may have also led to the depletion of histones in the LPON model. The expression levels of Histone 1 proteins and high mobility group nucleosome-binding domain-containing protein 3 isoform HMGN3a (HMGN3), a regulator of chromatin structure, can have a large effect on the transcription profile of cells. Traditionally, these proteins are seen as transcriptional repressors although in some circumstances they can act as activators [30, 31]. In our model, a possible explanation for the diminished histones is that it reflects the overall reduction in nucleosomes to allow DNA transcription process [32].

Although chromatin has been found to have an increasingly complex role in DNA damage response and cellular function, it appears that one of its basic roles remains to be physical shielding of DNA from damage [33]. It has been shown that depletion of histones including H1, can enhance the rate of DNA damage resulting from ionising radiation [34]. Thus, it is conceivable that histone depleted regions of DNA with LPON could also be susceptible to insult by ROS leading to further depletion. Indeed, hepatocytes in NAFLD are associated with DNA damage and lack of cell cycle progression beyond G1/S phase [35]. In addition, ROS-induced DNA damage has been shown to contribute to steatoapoptosis in a high fat diet mouse model of NAFLD [36].

Therefore, it is possible that chromatin down regulation with LPON represents an adaptation to increased requirements for DNA transcription in order to cope with the increased metabolic demand. However, in a metabolic milieu characterised by increased ROS formation, this most likely predisposes to ROS-induced DNA damage. It is also possible that this vicious cycle will increase the risk of mutations and epigenetic modulations hence the risk for development of hepatocellular carcinoma in a background of steatohepatitis [37–39].

## Conclusion

**Fig. 3:**
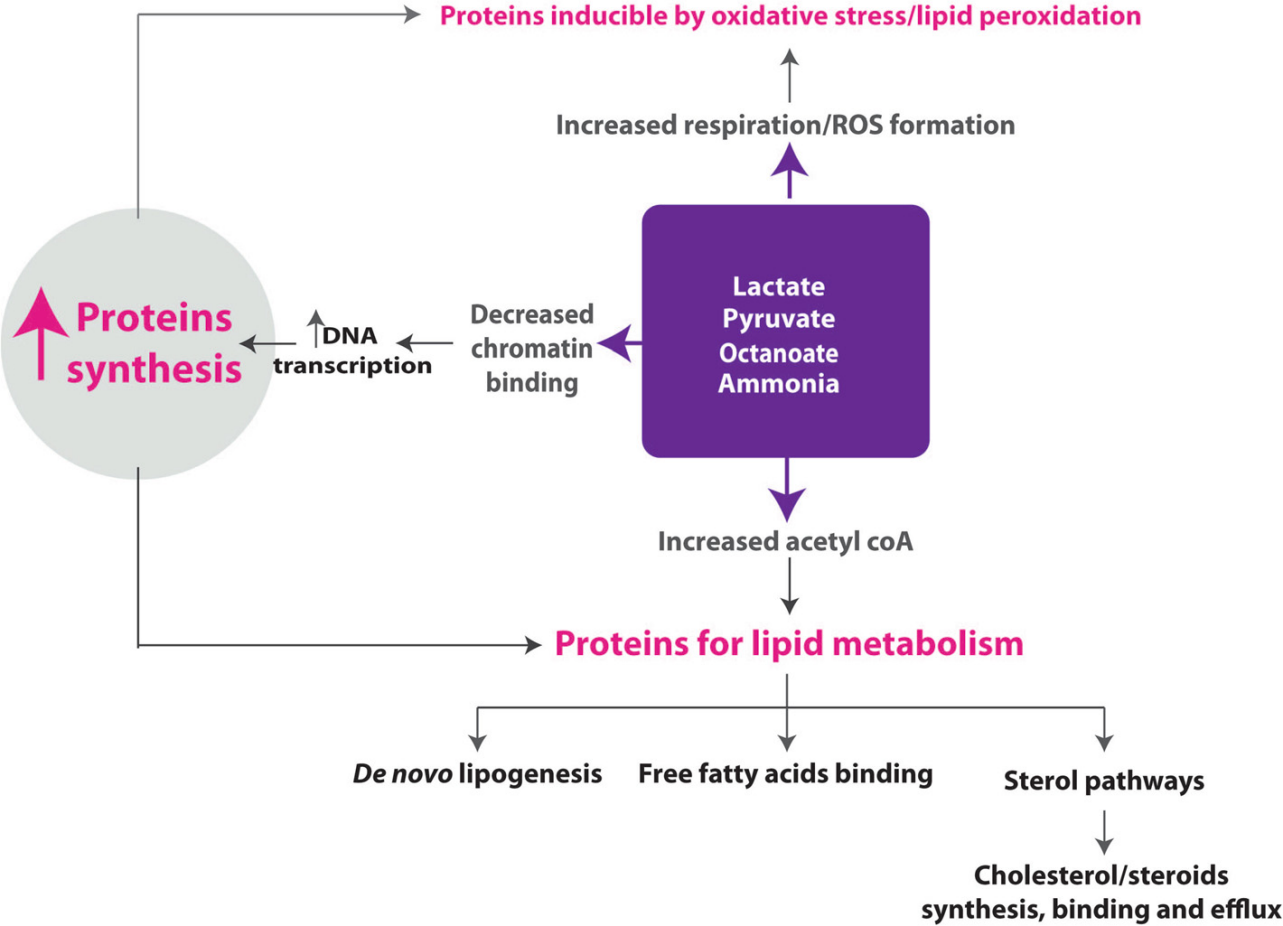
Increased protein synthesis following a surge of energy substrates with concomitant rise in reactive oxygen species. Our proteomic analysis suggests that increased energy substrates instigates processes that limit lipotoxicity potentially to curb oxidative stress. This involves diversion towards non-oxidative pathways with concomitant rise in the efflux of lipid from the liver. The associated changes in histones are most likely to represent an adaptation to increased transcription but whether such alterations can increase DNA susceptibility to further oxidative stress damage should be explored

In conclusion, the present study has given us a hepatocyte perspective of the pathogenesis of NAFLD by distilling key deranged molecular functions from enriched protein sets. The picture generated from our proteomic analysis suggests that the surge of energy substrates instigates processes that limit lipotoxicity and curb further oxidative stress (Fig. 3). This involves diversion towards non-oxidative pathways with concomitant rise in the efflux of lipid from the liver. This is paralleled by mechanisms geared to cope with increased oxidative stress. The associated changes in histones and potentially the nucleosomes appear to be significant and deserve further study. Although these changes most likely represent an adaptation to increased transcription, the role of fatty acid and/or oxidative stress in mediating these changes and whether such alterations can increase DNA susceptibility to further oxidative stress damage should be explored particularly in the context of hepatocarcinogenesis.

## Abbreviations

AKR1C1: aldo-keto reductase family 1 member C1
ALB: albumin
APOA1: apolipoprotein A1
APOE: apolipoprotein E
DDT: dithiothreitol
ER: endoplasmic reticulum
FABP: fatty acid binding protein
FCS: foetal calf serum
FDR: false discovery rate
FFA: free fatty acid(s)
HDLBP: high density lipoprotein binding protein
HNF4A: hepatocyte nuclear factor 4-alpha
IGF2R: insulin-like growth factor 2 receptor
LPON: lactate pyruvate octanoate and ammonia
MEME: minimum essential medium Eagle
NAFLD: non-alcoholic fatty liver disease
NASH: non-alcoholic steatohepatitis
NRF2: NF-E2-related factor-2
PBS: phosphate buffered saline
PLIN2: perlipin 2
ROS: reactive oxygen species
TCA: tricarboxylic acid cycle
TPCK: L-1-Tosylamide-2-phenylethyl chloromethyl ketone
TRiC: TCP-1 Ring Complex
UPR: unfolded protein response
